# A 45 nm CMOS Avalanche Photodiode with 8.4-GHz Bandwidth

**DOI:** 10.3390/mi11010065

**Published:** 2020-01-07

**Authors:** Wenhao Zhi, Qingxiao Quan, Pingping Yu, Yanfeng Jiang

**Affiliations:** 1 School of IoT Engineering, Jiangnan University, Wuxi 214122, China; 2 Haobang high-tech Co. LTD, Wuxi 214074, China

**Keywords:** CMOS compatible technology, avalanche photodiode, SPICE model, bandwidth, high responsivity, silicon photodiode

## Abstract

Photodiode is one of the key components in optoelectronic technology, which is used to convert optical signal into electrical ones in modern communication systems. In this paper, an avalanche photodiode (APD) is designed and fulfilled, which is compatible with Taiwan Semiconductor Manufacturing Company (TSMC) 45-nm standard complementary metal–oxide–semiconductor (CMOS) technology without any process modification. The APD based on 45 nm process is beneficial to realize a smaller and more complex monolithically integrated optoelectronic chip. The fabricated CMOS APD operates at 850 nm wavelength optical communication. Its bandwidth can be as high as 8.4 GHz with 0.56 A/W responsivity at reverse bias of 20.8 V. Its active area is designed to be 20 × 20 μm^2^. The Simulation Program with Integrated Circuit Emphasis (SPICE) model of the APD is also proposed and verified. The key parameters are extracted based on its electrical, optical and frequency responses by parameter fitting. The device has wide potential application for optical communication systems.

## 1. Introduction

As one of the promising photoelectric sensors, avalanche photodiode (APD) breaks the limitations of electrical interconnects, which results in high-speed, dense, and low-power interconnects [1]. It has become one of the research hotspots in the field of optical communication in recent years [2]. Avalanche photodiodes are widely used in optical communication systems and optical interconnection equipment, such as local area network, chip-to-chip, and board-to-board interconnect [3]. As one of them, 850 nm optical interconnects are actively being investigated, because 850 nm can be easily available as light sources in the high-speed optical interconnects [4,5]. The monolithically integrated high speed 850 nm wavelength silicon APDs based on standard complementary metal–oxide–semiconductor (CMOS) technology are particularly attractive because of significant advantages in cost, power, and performance that CMOS technology brings [6].

However, the optical absorption coefficient of silicon is fairly low at 850 nm. Since in standard CMOS technology, the silicon substrate is thicker than the penetration depth of light, which generates a large number of carriers in the silicon substrate and diffuses around [6]. Secondly, the maximum support voltage is reduced as the CMOS technology shrinks, which limits the reverse bias voltage for the integrated APDs [7].

Several approaches have been proposed to overcome the deficiencies and improve the performance of CMOS silicon technology. In [8], Huang et al. fabricated a silicon photodiode in standard 0.18 μm CMOS technology. The basic structure of proposed photodiode is formed by multiple p-n diodes with shallow trench isolation (STI) between p and n region. The fabricated photodiode demonstrates the −3 dB bandwidth of 1.6 GHz and a high responsivity of 0.74 A/W. In order to reduce the limit of bandwidth, Lee [9] proposed a spatially modulated avalanche photodiode (SM-APD), which showed a bandwidth of 12 GHz and responsivity of 0.03 A/W. Iiyama et al. fabricated a triple-well structure Si photodiode with standard 0.18 μm CMOS process [10]. The N+ and P+ layers are alternatively arranged and then the electrodes are interdigital structure. The device shows a 10 GHz bandwidth with 0.05 A/W responsivity [10]. Deep N-well CMOS technology can greatly improve the electrical isolation performance between different circuit blocks, which is especially important for integrating RF-to-baseband mixed-mode circuits in a single chip. The device with Deep N-well structure can substantially increase the cut-off frequency. In the paper [11], Chou et al. used extra bias on the Deep N-well in standard CMOS technology, which achieved a high bandwidth (8.7 GHz) with a responsivity of 0.05 A/W under a 11.45 V bias.

In this paper, a P-well/Deep N-well APD based on 45 nm CMOS technology is proposed. The light current, dark current, responsivity, and photodetection frequency response are measured based on the fabricated APD device. The results show that the fabricated APD presents a high responsivity and a high bandwidth. The 8.4 GHz bandwidth is available at 850 nm with 0.56 A/W responsivity. Finally, the key parameters of APD are extracted from the frequency response. A SPICE model is established for future integrated circuit design and simulation.

## 2. Design and Analysis of CMOS Compatible Avalanche Photodiode (APD)

P-well/Deep N-well structure is considered to be the most suitable structure for fabricating CMOS photodiodes [12]. J. Goy et al. compared various photodiode structures, such as N-well/P-substrate structure, N+/P-substrate structure, and N+/P-well structure. The results indicated that the P-well/Deep N-well structure can improve the responsivity while reducing the parasitic capacitance [12].

**Figure 1:**
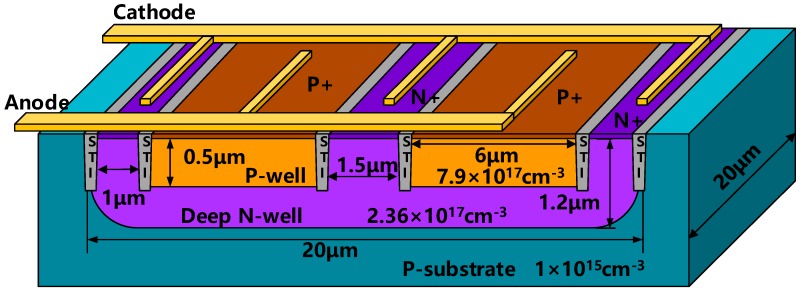
Structure of the designed complementary metal–oxide–semiconductor (CMOS) avalanche photodiode.

Two types of APDs, with different active areas, 20 × 20 μm^2^ and 50 × 50 μm^2^, are fabricated, separately. Figure 1 shows the schematic structure of the 20 × 20 μm^2^ CMOS APD device. The size of 50 × 50 μm^2^ device is proportional to the 20 × 20 μm^2^. The design is compatible with TSMC 45 nm standard CMOS technology without any process modification or special substrate. The APD is realized by vertical P-well/Deep N-well with shallow trench isolation (STI).

The contribution of slow diffusion photo-generated carriers in the P-substrate region can be excluded by the Deep N-well [13]. Moreover, the P-substrate is grounded or connected to a negative potential, which can effectively absorb slow diffusion photo-generated carriers. As a result, the P-well/Deep N-well shows a better performance in photodetection bandwidth than N-well/P-substrate photodiode.

When the reverse bias voltage is high, the electric field of the p-n junction increases rapidly. Because of curvature effect, the local electric field is increased, which makes the edge of the photodiode easily to breakdown [14]. It has a detrimental effect on the stability and performance of CMOS photodiodes. The most common method to prevent photodiode edge breakdown is to use a guard ring structure [15]. In this paper, STI with width of 0.15 μm is used as the guard ring (the junction depth is about 0.5 μm). The STI can improve the reverse bias by mitigating the premature edge breakdown during avalanche. A high reverse bias provides better avalanche gain and higher responsivity.

**Figure 2:**
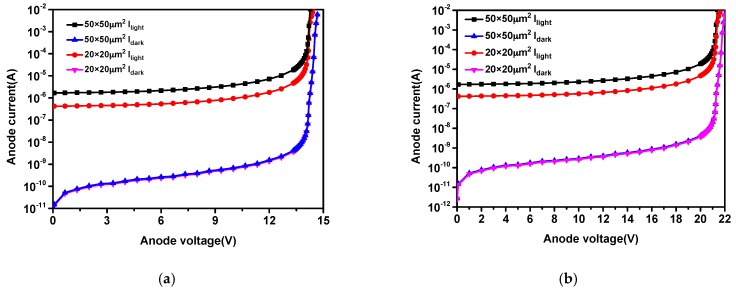
Electrical responses of the avalanche photodiodes (APDs) with different sizes (**a**) without shallow trench isolation (STI); (**b**) with STI.

To investigate its characteristics, a 100 μW, 850 nm, 10 Gb/s VCSEL modulated by Agilent E8257D signal generator is used as the light source. Figure 2 shows I-V characterizations of the APDs under light and dark environments, separately. All APDs show very low dark currents, which being less than 0.1 nA before the avalanche breakdown. Due to the influence of the STI structure, the avalanche breakdown voltage is increased from 14.5 V to 21.5 V. When the reverse bias approaches the avalanche breakdown voltage of 21.5 V, the dark current begins to increase sharply because of the occurrence of the avalanche breakdown. The 50 × 50 μm^2^ APD active area is larger than 20 × 20 μm^2^, so the photocurrent of 50 × 50 μm^2^ APD is also larger.

**Figure 3:**
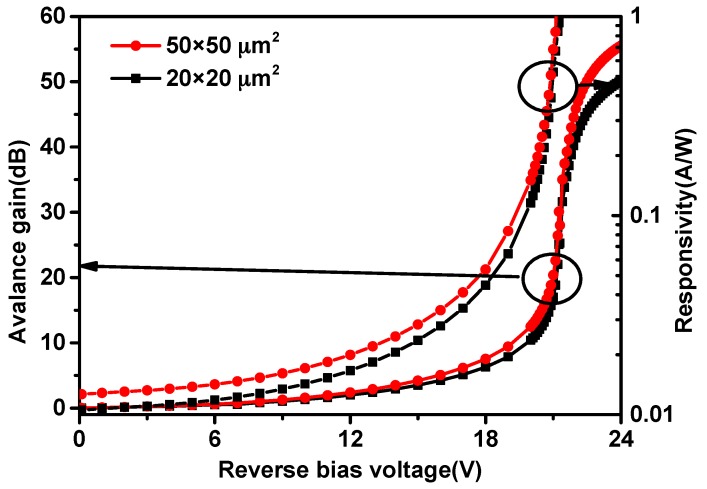
Optical responses of the APDs.

Responsivity is defined as the photocurrent per incident optical power, which is determined by the current under illumination minus the dark current [16]. Figure 3 shows the avalanche gain, and the responsivity obtained from the measured I-V characterization. The dark current will increases to the same level as the photocurrent when the avalanche breakdown occurs. In order to reduce the influence of the dark current noise, the operating point should be slightly less than 21.5 V. Considering all the related aspects, the operating point is set to be 20.8 V, and the gain is about 23 dB. Due to the STI structure, the reverse bias is significantly improved. As the reverse bias increases, the photocurrent and the responsivity of APDs are improved obviously. When the reverse bias voltage is 20.8 V, the responsivity of the APD with area of 50 × 50 μm^2^ is 0.59 A/W. On the same condition, the responsivity of the APD with area of 20 × 20 μm^2^ is 0.56 A/W.

**Figure 4:**
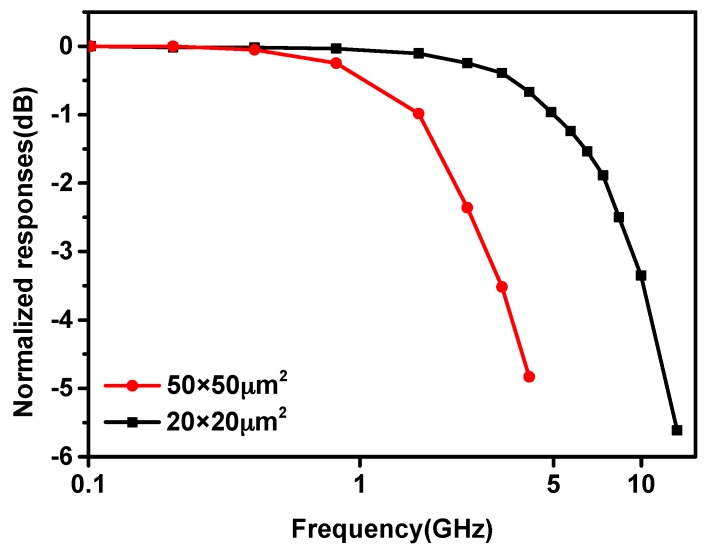
The frequency response of the fabricated APDs.

Figure 4 shows the frequency response of the two APDs with different active areas. The bandwidth of 50 × 50 μm^2^ is much lower than that of 20 × 20 μm^2^. With the increasing of the active area, the parasitic capacitance and the carrier transit time increase accordingly, which deteriorates the frequency property. The APD with active area of 20 × 20 μm^2^ shows a bandwidth of 8.4 GHz at a reverse bias of 20.8 V.

## 3. The SPICE Model of the CMOS APD

**Figure 5:**
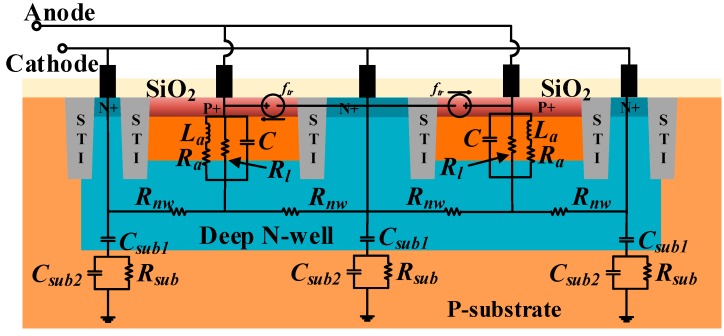
Simulation Program with Integrated Circuit Emphasis (SPICE) model of the CMOS APD.

In order to better understand the photodetection frequency response characterization of the CMOS APD, the SPICE model is set up in the section. We have adjusted and optimized the SPICE model proposed in reference [16] to fit the proposed structure in the paper. The values of the key parameters are extracted from the results of Figure 2, Figure 3 and Figure 4 by parameter fitting. For the parameter fitting, the initial value comes from the theoretical equation and then is manually modified. Figure 5 shows the updated SPICE model based on the detailed structure of the device. The active part is composed by an inductor and a resistor in series, a resistor in parallel and a capacitor. The capacitor C denotes the capacitance of the depletion region. Resistor *R_l_* denotes the resistance of the depletion region [17]. Inductor *L_a_* indicates the phase delay between the current and voltage caused by the impact ionization [17]. Series resistor *R_a_* indicates reverse saturation current and field-dependent velocity [17]. *R_nw_* indicates the Deep N-well resistance. R*_sub_* indicates the substrate resistance. *C_sub1_* denotes the capacitance between Deep N-well and P-substrate [18]. *R_sub_* and *C_sub2_* are caused by the parasitic effects of P-substrate [18]. The effect of the photo-generated slow carrier transit time is denoted by the current source *f_tr_* [16].

**Figure 6:**
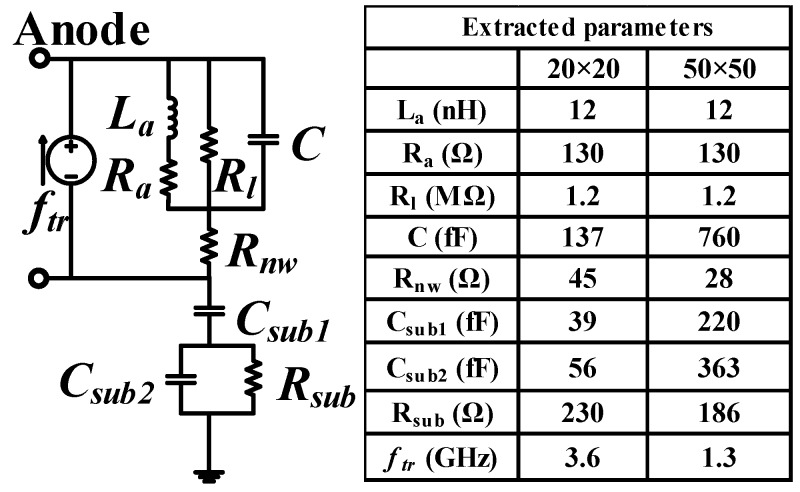
SPICE model and the extracted parameters of the APD.

Figure 6 shows the extracted parameter values for the simulation. The values of *L_a_*, *C*, and *R_nw_* are calculated by the following equations.

$$L_{a}=\tau_{a}/\left(2\alpha^{\prime }I_{0}\right),\;C=\varepsilon_{s}A/W_{D},\;R_{nw}\approx W_{d}/2A\varepsilon_{s}\nu_{s}$$

where $\tau_{a}$ is the transit time across the avalanche region, α’ is the derivative of the ionization coefficient with respect to the electric field, $I_{0}$ is the bias current, $\varepsilon_{s}$ is the semiconductor permittivity, *A* is the cross sectional area, $W_{D}$ is the depletion region width, $W_{d}$ is the drift region width, and $\nu_{s}$ is the saturation [19].

The *f_tr_* is estimated as $f_{tr}\approx \left(1/\left(2\pi \tau_{\mathrm{tr}}\right)\right)$, and $\tau_{tr}$ is expressed as $\tau_{tr}\approx 4L^{2}/\left(\pi^{2}D\right)$, where *L* is the diffusion length, *D* is the diffusion coefficient [20]. Later, these parameters will be re-corrected by the parameter fitting of the measured reflection coefficients and the frequency response.

**Figure 7:**
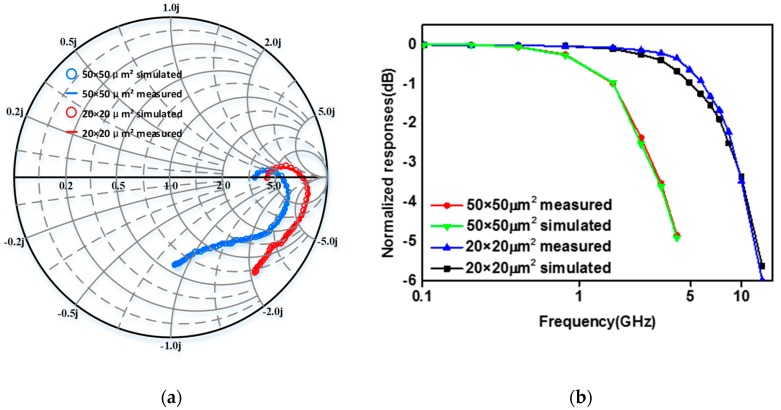
Comparison of the reflection coefficient and the frequency response between the measured and the simulated ones (**a**) reflection coefficients; (**b**) frequency response.

The reflection coefficients were measured by a vector network analyzer (Agilent E8362B) under a 100 μW, 850 nm, 10 Gb/s optical signal. From the measured reflection coefficients (shown in Figure 7a), Y-parameters and Z-parameters were calculated. *R_a_* and *R_l_* were extracted by the calculated Z-parameters. *R_a_* and *R_l_* were also re-corrected by parameter fitting. Then using ADS to perform parameter fitting of SPICE model to obtain the values of other parameters and manually modify them.

The extracted parameters for *R_l_*, *L_a_*, and *R_a_* are the same for all prepared devices. *R_l_* is defined as the voltage to current ratio near 0 V. The slope of the I-V characterization for all APD is shown in Figure 2, making *R_l_* the same for prepared devices. *L_a_* does not vary with device area at the same bias voltage, because the prepared APDs have the same avalanche multiplication characteristics based on the P-well/Deep N-well junction and guard ring as shown in Figure 2.

*R_a_* denotes the series resistance associated with the avalanche inductor *L_a_*, which determines the quality factor of the avalanche inductance [17]. It is not directly related to the device area. The junction capacitance *C* is proportional to the area. *C_sub1_* and *C_sub2_* are also proportional to the area. *R_nw_* and *R_sub_* do not change much because the increase in lateral resistance makes up for the decrease in vertical resistance.

Figure 7 shows the difference between experiment and simulation of the reflection coefficients and frequency response characterization, respectively. Based on the comparison shown in Figure 7, the simulation result based on the SPICE model coincides with the experiment ones, showing the accuracy of the proposed SPICE model.

Table 1 shows the comparison of the performance of various silicon photodetectors fabricated with standard CMOS technology. Our 20 × 20 μm^2^ CMOS APD shows the responsivity with 0.56 A/W and a photodetection bandwidth of 8.4 GHz at a reverse bias voltage of 20.8 V.

## 4. Conclusions

In this paper, an avalanche photodiode is designed and implemented based on 45 nm standard CMOS technology without any process modification. The fabricated CMOS APD shows a high response and high light detection bandwidth. Two types of CMOS APDs with different active areas are prepared, and their I-V characterization, photodetection frequency responses are examined. By reducing the active area from 50 × 50 μm^2^ to 20 × 20 μm^2^, the optical detection bandwidth of the prepared APD is increased to 8.4 GHz due to the decreased transit time, and the responsivity achieved 0.56 A/W. At the same time, the SPICE model of the fabricated CMOS APD device is set up for future circuit design and simulation. The key parameters based on the actual structure and the measurements are extracted. The simulation results show the accuracy of the proposed SPICE model. The proposed CMOS APDs are very useful for achieving high responsivity, and high speed 850 nm integrated optical receivers based on the standard CMOS technology.

Our future work will focus on reducing the bias voltage and power consumption of the device, while improving its photoelectric detection performance. Improving the light absorption, optimizing the doping concentration and doping depth can further improve the photoelectric detection performance of the device. Light absorption can be increased by adding an anti-reflection layer on the surface of the device. The optimization of doping concentration and doping depth requires more experiments to explore. On the other hand, optimizing the structure and parameters of the design to suit different wavelengths of photoelectric detection is also one of our future research directions.

## Figures and Tables

**Table 1:** The performances of various silicon photodetectors.

Parameters	Ref. [9]	Ref. [11]	Ref. [16]	Ref. [21]	This Work
**Technology**	0.13 μm	0.18 μm	0.13 μm	0.13 μm	45 nm
**Structure**	P+/N-well SM-APD	Multiple N+/P-sub APD	P+/N-well APD	N+/P-well APD	Double P-well/Deep N-well APD
**Area (μm^2^)**	5 × 5	50 × 50	10 × 10	30 × 30	20 × 20
**Bandwidth (GHz)**	12	8.7	7.6	3.5	8.4
**Responsivity (A/W)**	0.03	0.05	0.48	3.92	0.56
**Gain**	10.6	62.3	15.4	18.8	23
**Bias voltage (V)**	9.7	11.45	10.25	10	20.8
